# Association between dietary inflammatory index and cardiovascular‑kidney‑metabolic syndrome: evidence from NHANES 1999–2018

**DOI:** 10.1186/s12872-025-05091-y

**Published:** 2025-10-09

**Authors:** Feng Chen, Zhe Gu, Xiaogang Guo

**Affiliations:** 1 State Key Laboratory of Cardiovascular Disease, Arrhythmia Center, Fuwai Hospital, National Center for Cardiovascular Diseases, Chinese Academy of Medical Sciences and Peking Union Medical College, Beijing, 100037 China; 2 State Key Laboratory of Cardiovascular Disease, Department of Cardiology, Fuwai Hospital, National Center for Cardiovascular Diseases, Chinese Academy of Medical Sciences and Peking Union Medical College, Beijing, 100037 China

**Keywords:** Dietary inflammatory index, Cardiovascular‑kidney‑metabolic syndrome, NHANES, Risk stratification, Mortality

## Abstract

**Background:**

The dietary inflammatory index (DII) quantifies the inflammatory potential of an individual’s diet and has been linked to various chronic diseases. However, the association between DII and the severity of cardiovascular-kidney-metabolic (CKM) syndrome, as well as its impact on long-term mortality, remains insufficiently understood.

**Methods:**

We analyzed data from 17,412 adults enrolled in the National Health and Nutrition Examination Survey (NHANES) between 1999 and 2018. Dietary Inflammatory Index (DII) scores were derived from 24-hour dietary recall data, and CKM syndrome was categorized according to standardized staging criteria. Baseline characteristics were compared across DII quartiles and CKM stages. Multivariable logistic regression was used to assess the association between DII and advanced CKM (Stages 3–4), while Cox proportional hazards models estimated hazard ratios (HRs) for all-cause and cardiovascular mortality. Potential dose–response relationships were explored using restricted cubic spline analyses.

**Results:**

Participants with higher DII scores demonstrated less favorable sociodemographic and metabolic characteristics. Each one-point increment in DII was associated with a 6% increase in the odds of advanced CKM syndrome (adjusted odds ratio [OR]: 1.06; 95% confidence interval [CI]: 1.02–1.11). Compared to individuals in the lowest DII quartile, those in the highest quartile had a 36% greater Likelihood of advanced CKM. Over a median follow-up of 118 months, elevated DII scores were significantly linked to increased risks of all-cause mortality (hazard ratio [HR]: 1.073; 95% CI: 1.046–1.100) and cardiovascular mortality (HR: 1.073; 95% CI: 1.023–1.124), with dose–response relationships consistent with linearity. Subgroup analyses revealed that these associations were particularly pronounced among women (in relation to CKM severity), former smokers and individuals without hypertension (for all-cause mortality), and participants with higher educational attainment (for cardiovascular mortality).

**Conclusions:**

A pro-inflammatory dietary pattern, as indicated by higher DII scores, is independently associated with greater severity of CKM syndrome and elevated long-term mortality. These findings highlight the potential of dietary inflammation as a modifiable target for CKM prevention and management.

## Contributions to the literature

First, this study systematically examined the associations between dietary inflammatory potential, CKM syndrome severity, and long-term mortality in a nationally representative population. By addressing these related outcomes within a single framework, the analysis offers a broader understanding of how diet-related inflammation may be linked to disease burden and prognosis.

Second, we found that higher DII scores were associated with more advanced CKM stages and elevated risks of both all-cause and cardiovascular mortality. These associations persisted after adjusting for key demographic, lifestyle, and clinical factors, and showed generally linear trends.

Third, while the observational nature of the data limits causal interpretation, the findings suggest that dietary inflammatory potential may play a role in the development and progression of cardiometabolic and renal conditions. These results may contribute to ongoing efforts to refine dietary guidance and chronic disease prevention strategies at the population level.

## Introduction

The American Heart Association (AHA) has recently introduced the concept of cardiovascular-kidney-metabolic (CKM) syndrome, a clinical construct reflecting the interconnected progression of obesity, diabetes, chronic kidney disease (CKD), and cardiovascular disease (CVD) [1, 2]. Rather than isolated conditions, these entities frequently co-occur and interact biologically, synergistically amplifying the risk of adverse health outcomes. The prevalence of CKM syndrome continues to rise, fueled by population aging, the global surge in metabolic disorders such as diabetes and obesity, and suboptimal control of hypertension [3–5]. Given its complex pathophysiology and escalating public health burden, there is a critical need to identify reliable, accessible biomarkers that can aid in the early recognition and comprehensive risk stratification of individuals at risk for CKM-related complications.

Systemic inflammation is increasingly recognized as a central mechanism underlying the development and progression of chronic conditions, including CVD, CKD, and diabetes [6–8]. Among modifiable lifestyle factors, diet plays a pivotal role in regulating inflammation through its effects on gut microbiota composition, oxidative stress pathways, and metabolic homeostasis [9]. To quantify the inflammatory potential of diet, the Dietary Inflammatory Index (DII) was developed based on 45 dietary components—encompassing nutrients, food groups, and bioactive compounds—classified by their pro- or anti-inflammatory properties [10]. A growing body of epidemiological and clinical evidence has linked higher DII scores with increased risks of various chronic diseases, including cancer, stroke, hypertension, diabetes, and CVD [11–16]. For instance, a 20-year longitudinal study involving over 70,000 women demonstrated a positive association between pro-inflammatory diets and the incidence of type 2 diabetes [13]. Similarly, analyses from the National Health and Nutrition Examination Survey (NHANES) reported that youth with higher DII scores were more likely to have hypertension [12]. Furthermore, a prospective study in a cohort of older Chinese adults revealed that elevated DII scores were associated with greater risks of both incident CVD and cardiovascular mortality [17].

While pro-inflammatory diets have been linked to cardiometabolic and kidney dysfunction, and some studies have explored the association between the DII and CKM syndrome [18, 19], few have systematically assessed whether higher dietary inflammatory potential corresponds to greater CKM severity or examined its implications for long-term mortality. Using data from the NHANES 1999–2018, we investigated the association between dietary inflammatory potential and CKM syndrome, with a focus on disease severity and subsequent risks of all-cause and cardiovascular mortality.

## Materials and methods

### Study population

This study utilized data from the NHANES conducted between 1999 and 2018. NHANES, led by the National Center for Health Statistics (NCHS) in collaboration with the Centers for Disease Control and Prevention, is a nationally representative cross-sectional survey designed to assess the health and nutritional status of the non-institutionalized U.S. population. Participants were selected using a stratified, multi-stage cluster sampling approach to enhance representativeness.

**Fig. 1:**
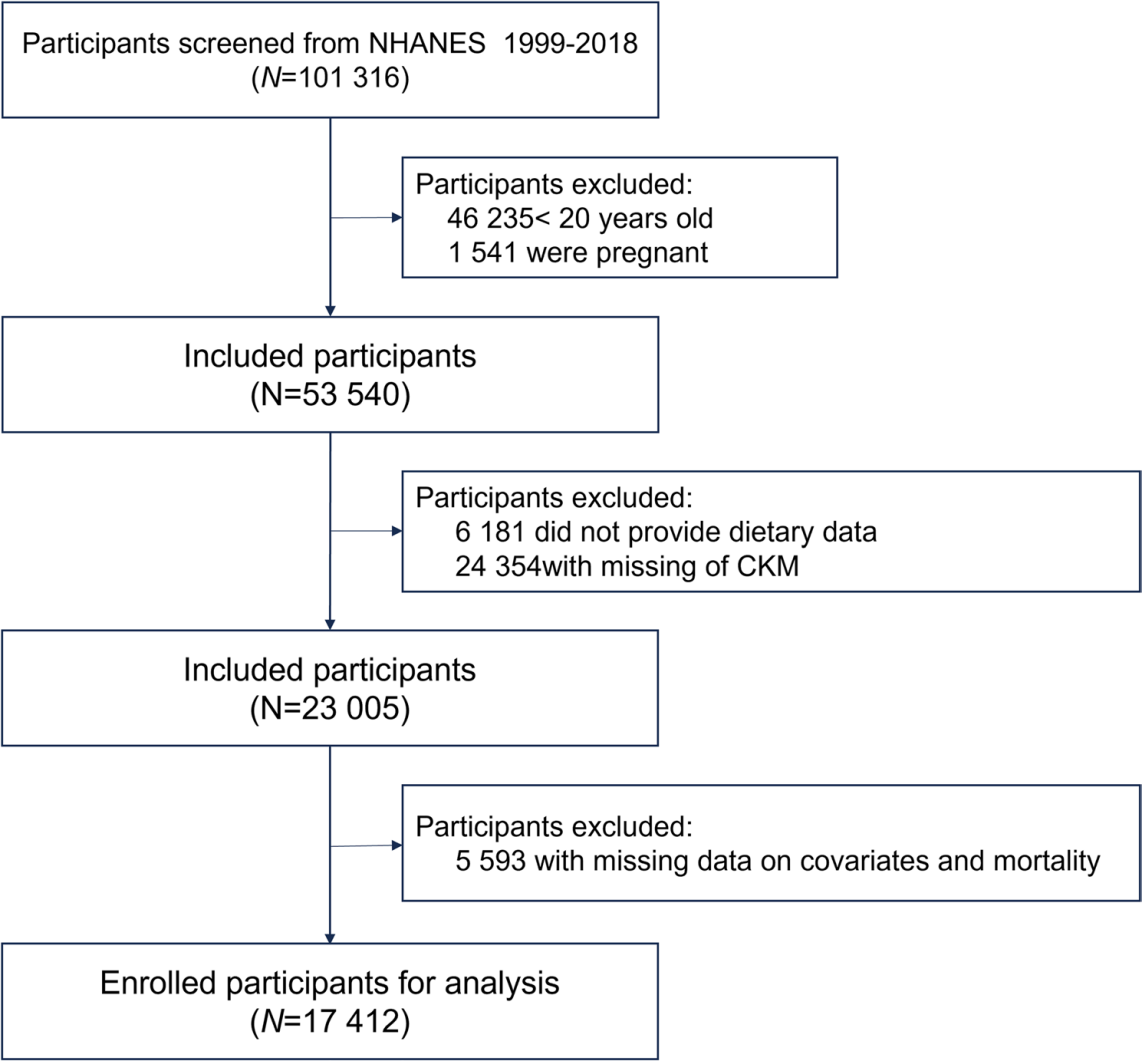
Flowchart of the study population. Abbreviations: NHANES National Health and Nutrition Examination Survey, CKM Cardiovascular- Kidney- Metabolic Syndrome

For this analysis, individuals were excluded if they met any of the following criteria: age under 20 years, pregnancy, missing dietary data, incomplete CKM assessment, unavailable covariate information, or loss to follow-up. After applying these exclusions, a total of 17,412 participants remained in the final dataset. The inclusion and exclusion criteria are illustrated in Fig. 1.

### Dietary inflammatory index (DII) assessment

The DII is a scoring system developed by Shivappa, designed to quantify the inflammatory potential of dietary components based on an extensive literature review [10]. The DII scores evaluate a diet’s overall inflammatory effect by considering its association with both pro-inflammatory markers—such as IL-1β, IL-4, IL-6, TNF-α, and C-reactive protein (CRP)—and the anti-inflammatory cytokine IL-10. It is calculated by summing the specific scores assigned to 45 food items and nutrients, generating an overall DII value. A positive DII score indicates a pro-inflammatory diet, whereas a negative DII score suggests an anti-inflammatory effect. Scores near zero imply minimal influence on inflammation.

In this study, the DII was derived from dietary intake data, incorporating 28 key nutrients, including macronutrients (carbohydrates, proteins, fats, alcohol, and dietary fiber), fatty acid subtypes (saturated, monounsaturated, polyunsaturated, omega-3, and omega-6), cholesterol, vitamins (A, B1, B2, B6, B12, C, D, E), and essential minerals or compounds (iron, magnesium, zinc, selenium, folic acid, beta-carotene, and caffeine), along with total caloric intake. Despite utilizing a subset of 28 nutrients instead of the full 45-component DII calculation, previous research has demonstrated that this approach maintains the index’s validity and predictive capability [10]. Dietary information was collected using one 24-hour dietary recall interview conducted at the Mobile Examination Center (MEC) as part of NHANES. Although two24-hour dietary recalls have been collected in NHANES since 2003, only one was available for earlier cycles (1999–2002). To ensure consistency across survey years, only the first day’s recall data were used in all analyses.

For analysis, the DII was treated as a continuous variable, and additionally categorized into quartiles: Q1 (DII scores ≤ 0.25), Q2 (0.25 < DII scores ≤ 1.76), Q3 (1.76 < DII scores ≤ 2.95), and Q4 (DII scores > 2.95), based on the distribution of DII scores.

### CKM syndrome stage assessment

CKM syndrome stages (0–4) were operationalized following the classification proposed by Aggarwal et al. [20]. with adaptations tailored to NHANES data. Stage 0 comprised individuals with normal weight (BMI 18.5–24.9) and low waist circumference (< 88 cm in women; <102 cm in men), absent any criteria for more advanced stages. Stage 1 included participants with overweight/obesity (BMI ≥ 25), elevated waist circumference (≥ 88 cm for women or ≥ 102 cm for men), or evidence of prediabetes—defined by fasting glucose 100–124 mg/dL or HbA1c 5.7–6.4%. Stage 2 was defined by the presence of metabolic risk factors, including hypertriglyceridemia (≥ 135 mg/dL), hypertension (self-reported history of hypertension, blood pressure ≥ 140/90 mmHg, or current antihypertensive treatment), diabetes (self-reported diagnosis, use of diabetes medications or insulin, or laboratory criteria [HbA1c ≥ 6.5%, fasting glucose ≥ 7.0 mmol/L, or random glucose ≥ 11.1 mmol/L]), metabolic syndrome (≥ 3 of the following: elevated waist circumference, low high density lipoprotein cholesterol (HDL) level [< 40 mg/dL or < 50 mg/dL for men or women, respectively], fasting serum triglycerides ≥ 150 mg/dL, elevated blood pressure [systolic blood pressure ≥ 130, diastolic blood pressure ≥ 80 mmHg, and/or use of blood pressure-lowering medications], or prediabetes), or moderate to high CKD risk (estimated glomerular filtration rate [eGFR] ≥ 60 mL/min/1.73 m² and urinary albumin-creatinine ratio [uACR] ≥ 30 mg/g, eGFR 45–59 mL/min/1.73 m² with uACR < 300 mg/g, or eGFR 30–44 mL/min/1.73 m² with uACR < 30 mg/g), based on Kidney Disease: Improving Global Outcomes (KDIGO) classification [21]. Stage 3 encompassed individuals with either very-high-risk CKD (eGFR 45–59 mL/min/1.73 m² with uACR ≥ 300 mg/g, eGFR 30–44 mL/min/1.73 m² with uACR ≥ 30 mg/g, or eGFR < 30 mL/min/1.73 m² regardless of uACR) or a predicted 10-year CVD risk ≥ 20%, estimated using the AHA PREVENT equations [22]. Stage 4 was assigned to those with self-reported cardiovascular disease, including coronary heart disease, myocardial infarction, angina, heart failure, or stroke. Renal risk stratification was conducted using the 2021 race-free CKD-EPI creatinine equation [23] and uACR. For analytic purposes, stages were further dichotomized into non-advanced (Stages 0–2) and advanced (Stages 3–4) CKM.

### Mortality outcomes

Mortality data were obtained from the Centers for Disease Control and Prevention and Linked to the NHANES dataset using unique participant identifiers. The follow-up period extended until December 31, 2019, with mortality status determined through national death records.

Causes of death were classified according to the Tenth Revision of the International Statistical Classification of Diseases and Related Health Problems (ICD-10). Cardiovascular mortality was defined as fatalities due to heart diseases (ICD-10 codes I00–I09, I11, I13, I20–I51) or cerebrovascular diseases (ICD-10 codes I60–I69). Each participant was followed from their initial NHANES examination until either the recorded date of death or the end of the follow-up period, whichever occurred first.

### Covariates

Demographic and health-related data were collected through in-home interviews, including age, sex, race/ethnicity, marital status, education level, poverty income ratio (PIR), alcohol consumption, and smoking status. PIR was dichotomized at 300%. Alcohol consumption was categorized into never drinkers (fewer than 12 drinks in their lifetime), former drinkers (at least 12 drinks in one year but not in the past year), and current drinkers (further classified as heavy drinkers, moderate drinkers, and light drinkers based on drinking patterns). Smoking status was categorized as never smokers (fewer than 100 cigarettes in their lifetime), former smokers (more than 100 cigarettes in their lifetime but not currently smoking), and current smokers (more than 100 cigarettes in their lifetime and currently smoking). Laboratory measurements, including BMI, cholesterol, triglycerides, HDL cholesterol, and serum creatinine, were considered potential confounders. Detailed measurement procedures are available on the NHANES website (https://wwwn.cdc.gov/nchs/nhanes/Default.aspx).

### Statistical analysis

All statistical analyses incorporated survey-specific weights and accounted for the stratified, multistage sampling design to ensure representativeness of the U.S. non-institutionalized population from 1999 to 2018. Continuous variables were presented as weighted means with standard errors, while categorical variables were summarized as unweighted counts and weighted percentages. Between-group differences were assessed using survey-weighted one-way ANOVA for continuous variables and survey-adjusted chi-square tests for categorical variables, with stratification by quartiles (Q1–Q4) of the DII scores and CKM syndrome stages. To evaluate the association between DII scores and advanced CKM syndrome, multivariate logistic regression models were constructed with varying levels of covariate adjustment. For logistic regression, CKM stages were grouped as non-advanced (Stages 0–2) versus advanced (Stages 3–4). The unadjusted (crude) model served as a reference, while Model 1 controlled for demographic factors, including age, gender, and race. Model 2 further adjusted for socioeconomic and lifestyle factors such as education level, marital status, poverty income ratio, alcohol consumption, smoking status, BMI, diabetes, and hypertension. The relationship between DII scores and the risks of cardiovascular and all-cause mortality among individuals across all CKM stages (0–4) was assessed using Cox proportional hazards models, applying the same adjustment framework. To examine potential nonlinear associations between DII scores and mortality risk, restricted cubic spline (RCS) analysis with 4 knots placed at the 5th, 35th, 65th, and 95th percentiles of the DII distribution was conducted. Additionally, subgroup analyses were performed across various stratifications, including age, gender, race, education level, marital status, poverty income ratio, alcohol consumption, smoking status, diabetes, and hypertension.

All statistical analyses were conducted using R software (version 4.4.1), with statistical significance defined as a two-tailed *P*-value < 0.05.

## Results

### Baseline characteristics of study participants stratified by DII scores quartiles

Table 1 presents the baseline characteristics of 17,412 participants categorized by quartiles of the DII scores. The mean age of the cohort was 47.24 ± 0.24 years, with males comprising approximately half the sample. Compared with individuals in the lowest DII scores quartile, those in the highest quartile tended to be older, female, non-Hispanic black, and less educated. They were also more likely to have lower income, be unmarried or not living with a partner, and smoke currently. With increasing DII, participants showed worsening metabolic indicators, including higher BMI, triglycerides, total cholesterol, and glycohemoglobin, along with lower HDL cholesterol. The rates of chronic diseases such as diabetes, hypertension, stroke, cardiovascular disease, heart failure, and chronic kidney disease were also higher in the groups with elevated DII scores. Notably, the weighted prevalence of advanced CKM syndrome (stages 3–4) increased progressively across quartiles of the DII score: 10.72% in Q1, 12.72% in Q2, 12.95% in Q3, and 13.39% in Q4, with an overall weighted prevalence of 12.85%.

**Table 1:** Survey-weighted characteristics according to the DII quartiles

Characteristic	Overall *n* = 17412	Q1 *n* = 4352	Q2 *n* = 4352	Q3 *n* = 4355	Q4 *n* = 4353	*P* value
Age category, *n* (%)						0.003
20–39	5680(35.87)	1419(34.56)	1369(34.13)	1446(37.04)	1446(38.12)	
40–59	5801(39.17)	1555(41.55)	1494(40.84)	1398(37.00)	1354(36.80)	
≥60	5931(24.96)	1378(23.89)	1489(25.02)	1511(25.96)	1553(25.08)	
Gender, *n* (%)						< 0.001
Female	8553(49.80)	1577(36.42)	1955(46.26)	2325(55.18)	2696(63.85)	
Male	8859(50.20)	2775(63.58)	2397(53.74)	2030(44.82)	1657(36.15)	
Race and ethnicity, *n* (%)						< 0.001
Mexican American	3008(7.69)	801(8.28)	795(7.97)	748(7.52)	664(6.84)	
Non-Hispanic Black	3353(9.74)	629(6.54)	751(8.70)	939(11.29)	1034(13.01)	
Non-Hispanic White	8322(71.68)	2198(74.77)	2090(71.70)	2032(70.44)	2002(69.33)	
Other	1370(5.88)	415(6.04)	371(6.32)	298(5.54)	286(5.56)	
Other Hispanic	1359(5.02)	309(4.36)	345(5.31)	338(5.21)	367(5.26)	
Education level, *n* (%)						< 0.001
< High school	2719(9.04)	547(6.87)	645(8.45)	694(9.22)	833(12.05)	
>High school	8987(59.85)	2644(68.62)	2385(63.51)	2132(56.77)	1826(48.72)	
High school	5706(31.11)	1161(24.51)	1322(28.04)	1529(34.01)	1694(39.23)	
Marital status, *n* (%)						< 0.001
Married or living with a partner	10,719(65.45)	2893(69.33)	2817(68.54)	2563(62.27)	2446(60.82)	
Not married nor living with a partner	6693(34.55)	1459(30.67)	1535(31.46)	1792(37.73)	1907(39.18)	
Poverty income ratio, *n* (%)						< 0.001
<300%	10,586(48.45)	2235(39.62)	2541(44.92)	2728(50.79)	3082(60.34)	
≥300%	6826(51.55)	2117(60.38)	1811(55.08)	1627(49.21)	1271(39.66)	
Smoking status, *n* (%)						< 0.001
Never	9228(52.66)	2369(54.40)	2342(54.05)	2300(51.80)	2217(49.97)	
Former	4506(25.98)	1254(29.94)	1203(27.18)	1072(24.78)	977(21.23)	
Now	3678(21.36)	729(15.66)	807(18.77)	983(23.42)	1159(28.79)	
Alcohol consumption, *n* (%)						< 0.001
Never	2300(10.51)	459(8.10)	544(10.48)	579(10.70)	718(13.20)	
Former	3056(14.46)	608(11.43)	693(13.30)	821(15.79)	934(17.92)	
Mild	5977(37.10)	1769(44.08)	1586(38.96)	1392(34.12)	1230(29.97)	
Moderate	2598(17.24)	636(16.80)	650(16.96)	686(18.13)	626(17.13)	
Heavy	3481(20.69)	880(19.59)	879(20.31)	877(21.27)	845(21.78)	
Diabetes, *n* (%)	3286(14.05)	709(12.22)	796(13.75)	870(14.61)	911(15.95)	< 0.001
Hypertension, *n* (%)	7432(37.62)	1734(35.30)	1826(38.16)	1874(36.96)	1998(40.48)	0.002
Coronary artery disease, *n* (%)	729(3.60)	180(3.70)	187(3.43)	170(3.45)	192(3.81)	0.82
Stroke, *n* (%)	634(2.75)	107(1.97)	152(2.70)	156(2.68)	219(3.80)	< 0.001
Cardiovascular disease, *n* (%)	1874(8.59)	386(7.24)	442(8.46)	476(8.58)	570(10.33)	< 0.001
Congestive heart failure, *n* (%)	532(2.33)	91(1.52)	129(2.40)	140(2.59)	172(2.91)	< 0.001
Chronic kidney disease, *n* (%)	2959(12.50)	577(9.88)	692(11.52)	792(13.41)	898(15.74)	< 0.001
CKM syndrome						< 0.001
CKM 0	1578(11.41)	471(13.35)	410(11.66)	355(10.78)	342(9.50)	
CKM 1	3177(20.29)	870(21.60)	775(19.38)	800(20.85)	732(19.15)	
CKM 2	9520(55.44)	2363(54.32)	2402(56.24)	2409(55.42)	2346(55.93)	
CKM 3	1104(3.63)	233(2.99)	282(3.65)	276(3.77)	313(4.21)	
CKM 4	2033(9.22)	415(7.74)	483(9.06)	515(9.18)	620(11.21)	
Advanced CKM syndrome	3137(12.85)	648(10.72)	765(12.72)	791(12.95)	933(15.42)	< 0.001
BMI (kg/m^2^)	28.75(0.08)	28.13(0.12)	28.61(0.15)	28.95(0.11)	29.41(0.16)	< 0.001
TG (mg/dl)	132.72(1.30)	128.71(1.97)	135.69(2.65)	137.43(2.85)	129.18(1.77)	0.01
TC (mg/dl)	195.61(0.51)	193.43(0.88)	196.30(0.78)	197.64(0.92)	195.27(1.09)	0.004
HDL-C (mg/dl)	53.59(0.21)	54.06(0.34)	53.72(0.35)	53.81(0.36)	52.67(0.34)	0.02
HbA1c (%)	5.57(0.01)	5.52(0.02)	5.59(0.02)	5.57(0.02)	5.59(0.01)	0.01
Scr (mg/dl)	0.88(0.00)	0.88(0.00)	0.88(0.01)	0.88(0.01)	0.88(0.01)	0.8
eGFR (ml/(min×1.73m^2^)	95.71(0.28)	96.60(0.41)	95.66(0.39)	95.40(0.44)	95.03(0.47)	0.02
DII	1.40(0.03)	−1.01(0.02)	1.04(0.01)	2.35(0.01)	3.63(0.01)	< 0.001

Continuous variables are presented as weighted mean (SE), and categorical variables are presented as absolute numbers (n) and survey-weighted percentage (%)

*Abbreviations BMI* Body mass index, *TC* Total cholesterol, *TG* Triglycerides, *Scr* Serum creatinine, *HDL-C* High density lipoprotein cholesterol, *eGFR* Estimated glomerular filtration rate, *DII* Dietary inflammatory index, *Q* Quartile, *SE* Standard error

### Baseline characteristics of study participants stratified by CKM syndrome stages

Table 2 summarizes the baseline characteristics of subjects across different stages of CKM syndrome. The overall mean DII score was 1.40 ± 0.03, with a progressive increase observed as CKM severity increased. Specifically, mean DII values rose from 1.14 ± 0.06 in Stage 0 to 1.32 ± 0.05 in Stage 1, 1.42 ± 0.03 in Stage 2, 1.61 ± 0.08 in Stage 3, and peaked at 1.64 ± 0.06 in Stage 4. In addition to poorer dietary quality, individuals at more advanced CKM stages were generally older, more likely to be male and non-Hispanic Black, had lower educational attainment, and were more often socioeconomically disadvantaged (PIR < 300%). They also had higher rates of smoking, alcohol consumption, and were more frequently married or cohabiting. Regarding metabolic and biochemical markers, individuals in advanced stages presented with elevated levels of BMI, triglycerides, total cholesterol, glycohemoglobin, and serum creatinine, along with significantly lower HDL-C levels, reflecting a clustering of adverse cardiometabolic risk factors.

**Table 2:** Baseline characteristics according to the cardiovascular-kidney-metabolic (CKM) syndrome stages

Characteristic	Overall *n* = 17412	CKM 0 *n* = 1578	CKM 1 *n* = 3177	CKM 2 *n* = 9520	CKM 3 *n* = 1104	CKM 4 *n* = 2033	Advanced CKM *n* = 3137	*P* value
Age category, *n* (%)								< 0.001
20–39	5680(35.87)	1109(66.51)	1742(53.49)	2746(30.59)	8(1.00)	75(4.71)	83(3.66)	
40–59	5801(39.17)	394(29.51)	1075(36.43)	3857(46.34)	51(6.11)	424(27.08)	475(21.16)	
>=60	5931(24.96)	75(3.98)	360(10.08)	2917(23.08)	1045(92.88)	1534(68.21)	2579(75.18)	
Gender, *n* (%)								< 0.001
Female	8553(49.80)	973(64.29)	1698(52.10)	4598(47.39)	421(43.06)	863(43.99)	1284(43.73)	
Male	8859(50.20)	605(35.71)	1479(47.90)	4922(52.61)	683(56.94)	1170(56.01)	1853(56.27)	
Race and ethnicity, *n* (%)								< 0.001
Mexican American	3008(7.69)	200(5.56)	627(9.99)	1813(8.17)	140(4.43)	228(3.63)	368(3.85)	
Non-Hispanic Black	3353(9.74)	242(7.64)	634(10.53)	1870(9.75)	202(10.50)	405(10.19)	607(10.28)	
Non-Hispanic White	8322(71.68)	863(76.19)	1342(67.42)	4278(70.89)	655(77.90)	1184(77.82)	1839(77.85)	
Other	1370(5.88)	169(6.27)	280(5.56)	791(6.21)	42(3.35)	88(5.11)	130(4.61)	
Other Hispanic	1359(5.02)	104(4.34)	294(6.50)	768(4.99)	65(3.82)	128(3.25)	193(3.41)	
Education level, *n* (%)								< 0.001
< High school	2719(9.04)	118(4.79)	352(6.59)	1513(9.29)	280(17.47)	456(14.85)	736(15.59)	
>High school	8987(59.85)	1021(69.72)	1889(65.88)	4802(58.68)	441(44.45)	834(47.53)	1275(46.66)	
High school	5706(31.11)	439(25.49)	936(27.53)	3205(32.04)	383(38.08)	743(37.62)	1126(37.75)	
Marital status, *n* (%)								< 0.001
Married or living with a partner	10,719(65.45)	859(59.64)	1966(65.57)	6044(66.93)	629(57.18)	1221(66.72)	1850(64.03)	
Not married nor living with a partner	6693(34.55)	719(40.36)	1211(34.43)	3476(33.07)	475(42.82)	812(33.28)	1287(35.97)	
Poverty income ratio, *n* (%)								< 0.001
<300%	10,586(48.45)	863(43.15)	1830(46.23)	5690(47.45)	787(65.02)	1416(59.39)	2203(60.98)	
≥300%	6826(51.55)	715(56.85)	1347(53.77)	3830(52.55)	317(34.98)	617(40.61)	934(39.02)	
Smoking status, *n* (%)								< 0.001
Never	9228(52.66)	982(60.51)	1898(57.18)	5064(52.46)	513(46.15)	771(36.75)	1284(39.41)	
Former	4506(25.98)	212(14.91)	636(23.55)	2389(25.91)	443(39.73)	826(40.04)	1269(39.95)	
Now	3678(21.36)	384(24.58)	643(19.27)	2067(21.64)	148(14.11)	436(23.21)	584(20.64)	
Alcohol consumption, *n* (%)								< 0.001
Never	2300(10.51)	189(10.04)	360(9.32)	1251(10.30)	218(18.96)	282(11.67)	500(13.73)	
Former	3056(14.46)	121(6.84)	365(10.34)	1576(14.13)	345(29.06)	649(29.16)	994(29.13)	
Mild	5977(37.10)	558(36.69)	1090(35.65)	3190(37.02)	427(42.37)	712(39.27)	1139(40.15)	
Moderate	2598(17.24)	336(23.01)	595(20.87)	1425(16.83)	65(5.23)	177(9.33)	242(8.17)	
Heavy	3481(20.69)	374(23.42)	767(23.83)	2078(21.72)	49(4.38)	213(10.57)	262(8.82)	
Diabetes, *n* (%)	3286(14.05)	0(0.00)	0(0.00)	1906(16.12)	563(51.95)	817(35.03)	1380(39.81)	< 0.001
Hypertension, *n* (%)	7432(37.62)	0(0.00)	0(0.00)	4993(50.48)	885(80.21)	1554(72.93)	2439(74.98)	< 0.001
Coronary heart disease, *n* (%)	729(3.60)	0(0.00)	0(0.00)	0(0.00)	0(0.00)	729(38.98)	729(27.98)	< 0.001
Stroke, *n* (%)	634(2.75)	0(0.00)	0(0.00)	0(0.00)	0(0.00)	634(29.81)	634(21.39)	< 0.001
Cardiovascular disease, *n* (%)	1874(8.59)	0(0.00)	0(0.00)	0(0.00)	0(0.00)	1874(93.12)	1874(66.82)	< 0.001
Congestive heart failure, *n* (%)	532(2.33)	0(0.00)	0(0.00)	0(0.00)	0(0.00)	532(25.22)	532(18.10)	< 0.001
Chronic kidney disease, *n* (%)	2959(12.50)	0(0.00)	0(0.00)	1465(13.03)	628(54.93)	866(35.63)	1494(41.08)	< 0.001
BMI (kg/m^2^)	28.75(0.08)	21.85(0.06)	28.20(0.12)	30.15(0.11)	28.39(0.23)	30.16(0.23)	29.66(0.17)	< 0.001
TG (mg/dl)	132.72(1.30)	72.62(0.69)	81.70(0.68)	159.21(2.00)	150.90(6.31)	152.93(3.27)	152.35(2.81)	< 0.001
TC (mg/dl)	195.61(0.51)	179.02(1.04)	188.25(0.96)	203.51(0.71)	191.76(1.58)	186.34(1.31)	187.87(1.06)	< 0.001
HDL-C (mg/dl)	53.59(0.21)	62.15(0.52)	56.85(0.41)	51.26(0.23)	51.48(0.52)	50.74(0.57)	50.95(0.42)	< 0.001
HbA1c (%)	5.57(0.01)	5.12(0.01)	5.29(0.01)	5.64(0.01)	6.21(0.06)	6.02(0.04)	6.08(0.03)	< 0.001
Scr (mg/dl)	0.88(0.00)	0.81(0.00)	0.84(0.00)	0.86(0.00)	1.26(0.05)	1.07(0.02)	1.12(0.02)	< 0.001
eGFR (ml/(min×1.73m^2^)	95.71(0.28)	105.14(0.51)	102.65(0.48)	96.27(0.32)	65.39(0.86)	77.32(0.60)	73.95(0.55)	< 0.001
DII	1.40(0.03)	1.14(0.06)	1.32(0.05)	1.42(0.03)	1.61(0.08)	1.64(0.06)	1.63(0.05)	< 0.001

Continuous variables are presented as weighted mean (SE), and categorical variables are presented as absolute numbers (n) and survey-weighted percentage (%)

*Abbreviations* *BMI* Body Mass Index, *TC* Total Cholesterol, *TG* Triglycerides, *Scr* Serum Creatinine, *HDL-C* High Density Lipoprotein Cholesterol, *eGFR* Estimated Glomerular Filtration Rate, *DII* Dietary Inflammatory Index, *Q* Quartile, *SE* Standard Error

### Association of DII scores with advanced CKM syndrome

**Fig. 2:**
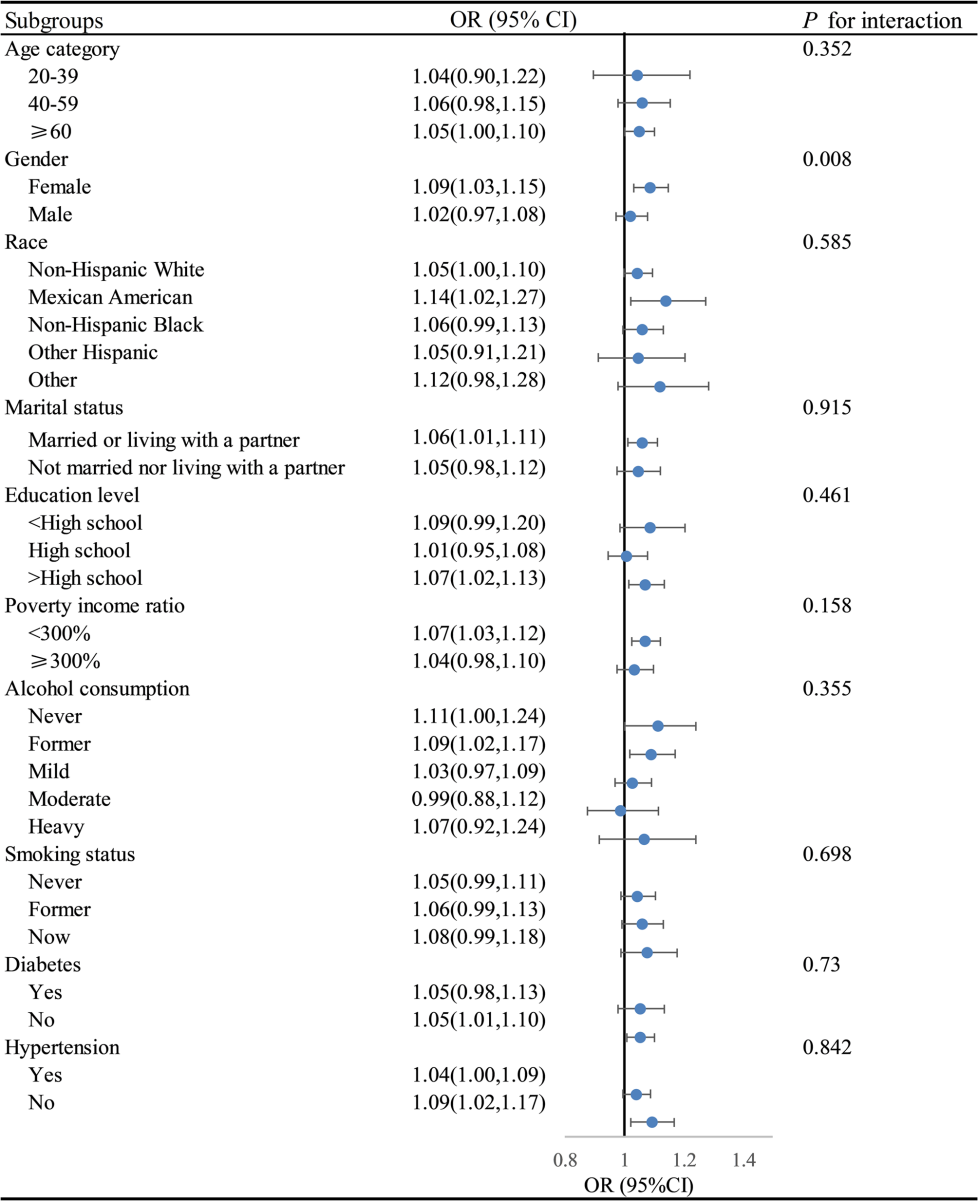
Subgroup analysis of the associations DII index with advanced CKM syndrome. Footnote: Adjusted for sex, age, race, education level, marital status, Poverty income ratio, alcohol consumption, smoking status, BMI, hypertension, and diabetes. Abbreviations: OR, odds ratio; CI, Confidence interval; DII, Dietary inflammatory index; CKM, Cardiovascular-kidney-metabolic

As shown in Table 3, higher DII scores were significantly associated with increased odds of advanced CKM syndrome. In the fully adjusted model (Model 2), each one-unit increase in DII was associated with a 6% higher risk (OR = 1.06, 95% CI: 1.02–1.11; *P* = 0.004). Compared with the lowest quartile, the adjusted odds ratios (95% CIs) for the second, third, and highest quartiles were 1.14 (0.95–1.36), 1.12 (0.92–1.36), and 1.36 (1.13–1.65), respectively, with a significant linear trend (*P* for trend < 0.001). In stratified analyses (Fig. 2), the association appeared stronger among females (OR = 1.09, 95% CI: 1.03–1.15) than males (OR = 1.02, 95% CI: 0.97–1.08), and a significant interaction by sex was observed (P for interaction = 0.008). No significant interactions were found for other stratifying variables, supporting the overall robustness of the association.

**Table 3:** Multivariate regression analysis of DII with advanced CKM syndrome

	Crude Model		Model 1		Model 2	
	COR (95% CI)	*P*-value	AOR (95% CI)	*P*-value	AOR (95% CI)	*P*-value
Continuous DII	1.08(1.05,1.12)	< 0.001	1.14(1.10,1.19)	< 0.001	1.06(1.02,1.11)	0.004
DII category						
Quartile 1	ref	ref	ref	ref	ref	ref
Quartile 2	1.28(1.08,1.50)	0.004	1.28(1.08,1.50)	0.004	1.14(0.95,1.36)	0.16
Quartile 3	1.39(1.18,1.65)	< 0.001	1.39(1.18,1.65)	< 0.001	1.12(0.92,1.36)	0.26
Quartile 4	1.92(1.62,2.29)	< 0.001	1.92(1.62,2.29)	< 0.001	1.36(1.13,1.65)	0.001
* P* for trend		< 0.001		< 0.001		0.004

Crude model, unadjusted; Model 1, adjusted for age, gender, race and ethnicity; Model 2, adjusted for age, gender, race, education level, marital status, poverty income ratio, alcohol consumption, smoking status, BMI, hypertension, and diabetes

*Abbreviations* *COR* Crude odds ratio, *AOR* Adjusted odds ratio, *CI* Confidence interval, *DII* Dietary inflammatory index, *CKM* Cardiovascular-kidney-metabolic

### Association of DII scores with mortality among CKM syndrome

During a median follow-up of 118 months, 2,679 of 17,412 participants with CKM syndrome (15.39%) died from any cause, and 850 (4.88%) died from cardiovascular causes. In the fully adjusted Cox model (Model 2), each one-point increase in DII score was significantly associated with a higher risk of both all-cause mortality (HR = 1.073; 95% CI: 1.046–1.100; *P* < 0.001) and cardiovascular mortality (HR = 1.073; 95% CI: 1.023–1.124; *P* = 0.003) (Table 4). When DII scores were divided into quartiles, those in the highest quartile exhibited a 31.4% higher risk of all-cause mortality and a 36.2% higher risk of cardiovascular mortality compared with the lowest quartile. A statistically significant dose–response trend was observed for both outcomes (*P* for trend < 0.001 for all-cause mortality; *P* for trend = 0.011 for cardiovascular mortality).

**Table 4:** Regression analysis of DII and all-cause mortality, cardiovascular mortality among CKM syndrome

	Crude Model		Model 1		Model 2	
	HR (95% CI)	*P*-value	HR (95% CI)	*P*-value	HR (95% CI)	*P*-value
All-cause mortality						
Continuous DII	1.113(1.084,1.142)	< 0.001	1.113(1.084,1.142)	< 0.001	1.073(1.046,1.100)	< 0.001
DII category						
Quartile 1	ref	ref	ref	ref	ref	ref
Quartile 2	1.331(1.152,1.539)	< 0.001	1.347(1.165,1.557)	< 0.001	1.224(1.060,1.414)	0.006
Quartile 3	1.489(1.303,1.703)	< 0.001	1.629(1.431,1.853)	< 0.001	1.304(1.140,1.492)	< 0.001
Quartile 4	1.610(1.408,1.840)	< 0.001	1.802(1.569,2.070)	< 0.001	1.314(1.146,1.508)	< 0.001
* P* for trend		< 0.001		< 0.001		< 0.001
Cardiovascular mortality						
Continuous DII	1.102(1.053,1.153)	< 0.001	1.155(1.106,1.207)	< 0.001	1.073(1.025,1.124)	0.003
DII category						
Quartile 1	ref	ref	ref	ref	ref	ref
Quartile 2	1.196(0.950,1.506)	0.127	1.294(1.018,1.645)	0.036	1.167(0.918,1.483)	0.207
Quartile 3	1.285(1.025,1.611)	0.030	1.548(1.248,1.919)	< 0.001	1.204(0.953,1.520)	0.120
Quartile 4	1.586(1.282,1.963)	< 0.001	1.910(1.522,2.397)	< 0.001	1.362(1.069,1.735)	0.013
* P* for trend		< 0.001		< 0.001		0.011

Crude model, unadjusted; Model 1, adjusted for sex, age, race and ethnicity; Model 2, adjusted for sex, age, race, education level, marital status, Poverty income ratio, alcohol consumption, smoking status, BMI, hypertension, and diabetes

*Abbreviations* *HR* Hazard ratio, *CI* Confidence interval, *DII* Dietary inflammatory index

**Fig. 3:**
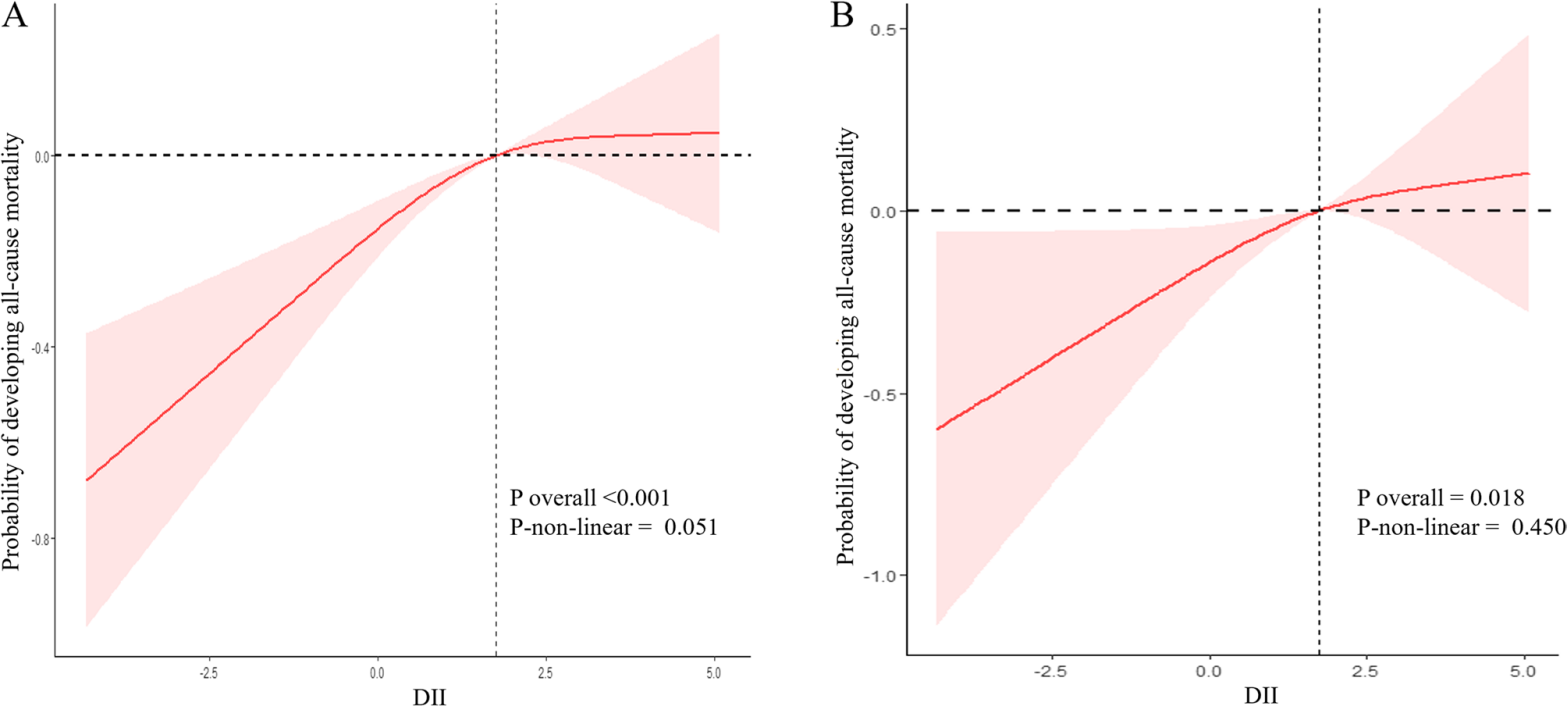
Restricted cubic spline curves showing the association of DII with the risk of all-cause mortality (**A**) and cardiovascular mortality (**B**) among CKM syndrome. Footnote: Adjusted for sex, age, race, education level, marital status, Poverty income ratio, alcohol consumption, smoking status, BMI, hypertension, and diabetes. Abbreviations: DII, Dietary inflammatory index; CKM, Cardiovascular-kidney-metabolic

**Fig. 4:**
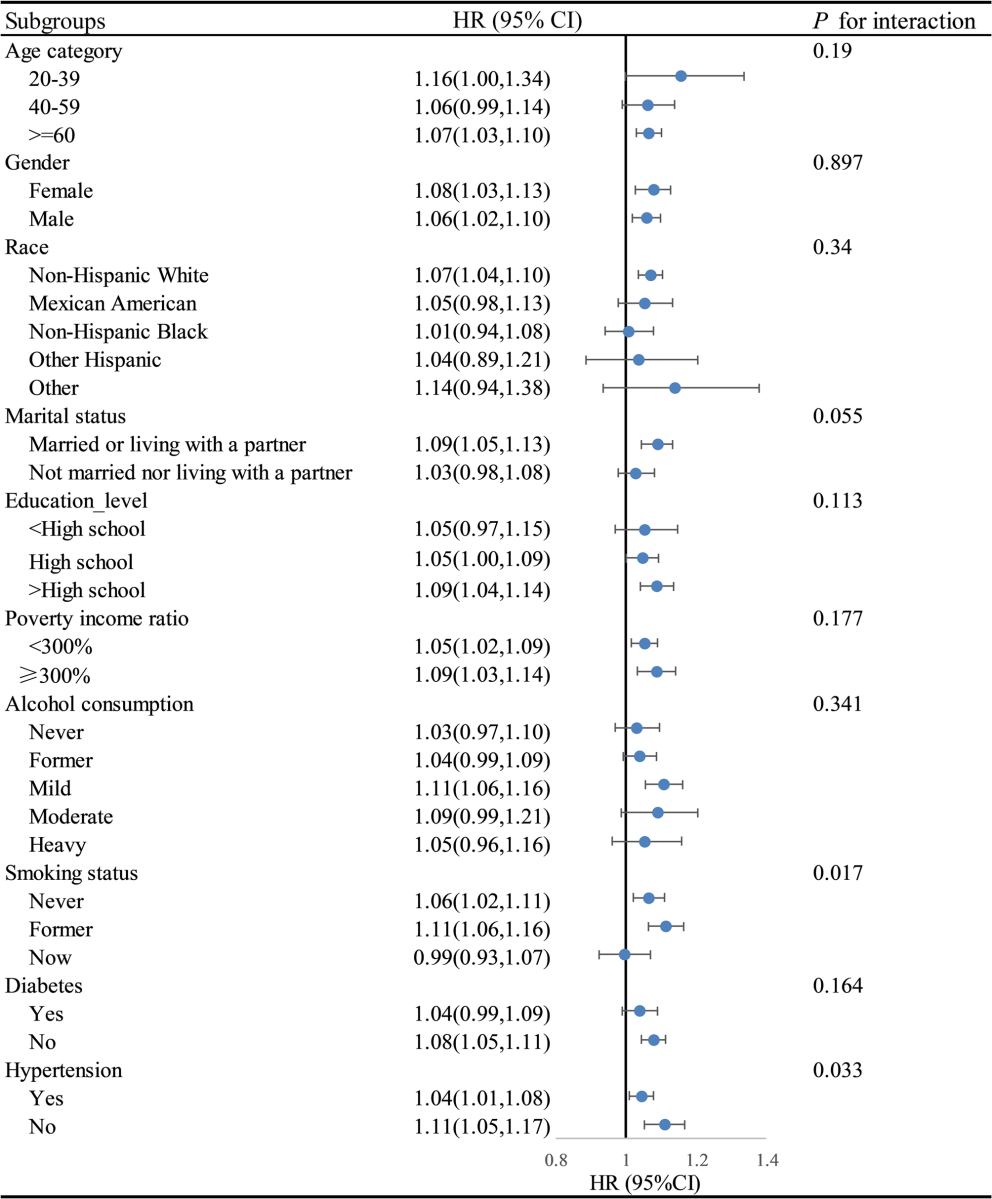
Subgroup analysis of the associations DII index with and all-cause mortality among CKM syndrome. Footnote: Adjusted for sex, age, race, education level, marital status, Poverty income ratio, alcohol consumption, smoking status, BMI, hypertension, and diabetes. Abbreviations: HR, Harzard ratio; CI, Confidence interval; DII, Dietary inflammatory index; CKM, Cardiovascular-kidney-metabolic

**Fig. 5:**
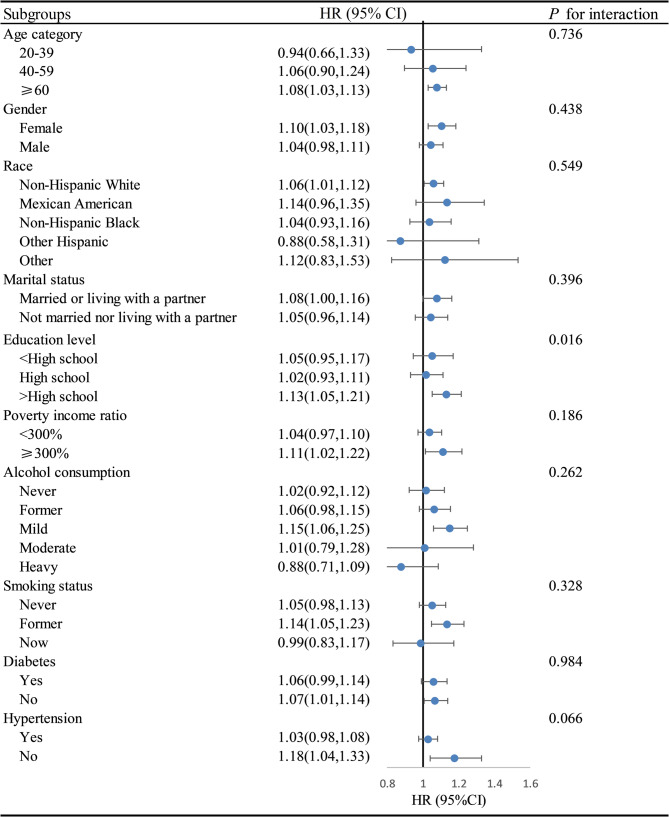
Subgroup analysis of the associations DII with and cardiovascular mortality among CKM syndrome. Footnote: Adjusted for sex, age, race, education level, marital status, Poverty income ratio, alcohol consumption, smoking status, BMI, hypertension, and diabetes. Abbreviations: HR, Harzard ratio; CI, Confidence interval; DII, Dietary inflammatory index; CKM, Cardiovascular-kidney-metabolic

Further analysis using restricted cubic spline models revealed no significant evidence of non-linearity in the association between DII scores and either all-cause (*P* = 0.051) or cardiovascular mortality (*P* = 0.450), indicating that a linear model adequately captures these relationships (Fig. 3). Subgroup analyses revealed significant interactions between DII scores and smoking status as well as hypertension in relation to all-cause mortality, and between DII scores and education level for cardiovascular mortality. Specifically, the association with all-cause mortality was stronger among former smokers than among never or current smokers, and more pronounced in individuals without hypertension compared to those with hypertension (Fig. 4). In terms of cardiovascular mortality, the relationship was more evident in participants with education beyond high school versus those with a high school education or less (Fig. 5).

## Discussion

In this nationally representative study, we identified a strong and graded association between higher DII scores and both the severity of CKM syndrome and long-term mortality risk. Specifically, individuals with more pro-inflammatory diets were significantly more likely to exhibit advanced CKM stages, and were at heightened risk for all-cause and cardiovascular death, independent of conventional risk factors. These findings extend prior research linking diet-induced inflammation to cardiometabolic dysfunction and underscore the potential utility of DII as an integrative marker of multisystem metabolic stress.

Prior research has established a positive association between the DII and CKM syndrome or its individual components, including cardiometabolic syndrome and CKD [18, 19]. However, most of these studies were limited by relatively small sample sizes, absence of disease severity stratification, and lack of longitudinal outcome data. As a result, the prognostic role of dietary inflammation within CKM progression and mortality remains inadequately characterized. Our study addresses this critical gap by leveraging a large, nationally representative cohort with extended follow-up to demonstrate that higher dietary inflammatory potential is independently and dose-dependently associated with more severe CKM syndrome and elevated risks of all-cause and cardiovascular mortality. These findings advance the understanding of diet-induced inflammation as a key factor not only in CKM development but also in its progression and prognosis. They further highlight the potential value of incorporating the DII into broader CKM risk stratification frameworks. While emerging indices such as the Atherogenic Index of Plasma (AIP), Triglyceride-Glucose (TyG) index, and the American Heart Association’s Life’s Essential 8 (LE8) score capture distinct aspects of lipid metabolism, insulin resistance, and lifestyle health, they do not directly reflect inflammatory dietary exposures [24–27]. The DII complements these tools by targeting a mechanistic pathway—chronic low-grade inflammation—that is increasingly recognized as a unifying driver of CKM pathology. Rather than serving as competing metrics, these indices may offer synergistic insights when integrated, especially for identifying high-risk individuals with overlapping metabolic and behavioral vulnerabilities. This approach is particularly relevant in the post-COVID-19 era, where the interplay between metabolic dysfunction and immune dysregulation has become increasingly apparent [28].

The biological plausibility of our findings is supported by multiple mechanistic pathways linking pro-inflammatory diets to development and progression of CKM syndrome. Diets rich in saturated fats, refined carbohydrates, and other pro-inflammatory components disrupt glucose and lipid metabolism, elevate oxidative stress, and promote chronic inflammation. These effects are mediated, in part, through endoplasmic reticulum stress and activation of toll-like receptors (TLR2 and TLR4), which trigger NF-κB and JNK signaling pathways [29]. These metabolic insults contribute to hallmark features of CKM, including insulin resistance, endothelial dysfunction, and renal injury [30–34]. Furthermore, diet-driven alterations in gut microbiota composition and intestinal barrier integrity may promote systemic endotoxemia, further amplifying inflammation and organ damage [35–37].

Our subgroup analyses further revealed notable heterogeneity in the relationship between dietary inflammation and mortality outcomes. The association with all-cause mortality was more prominent among former smokers and individuals without hypertension, whereas the link with cardiovascular mortality was particularly evident in participants with higher educational attainment. These variations may be explained by differences in baseline inflammatory profiles, dietary assessment accuracy, or underlying metabolic reserve. For example, individuals with higher education levels may adhere more consistently to health-conscious behaviors and provide more reliable dietary data, thereby allowing the DII to more precisely reflect true dietary inflammatory exposure [38]. Former smokers, despite quitting, may retain a residual pro-inflammatory milieu, potentially heightening their susceptibility to the adverse effects of a pro-inflammatory diet. In contrast, current smokers—who already exhibit chronically elevated inflammatory markers—may experience a ceiling effect that masks additional dietary contributions [39–43]. Similarly, in individuals without hypertension, the absence of established vascular inflammation may unmask a clearer association between diet and mortality risk, whereas in those with existing hypertension-related inflammation, the incremental impact of diet may be attenuated or less discernible [44–47].

Key strengths of this study include the use of a large, nationally representative cohort with extended follow-up; the application of standardized, comprehensive criteria to define CKM multimorbidity; and rigorous adjustment for a wide spectrum of demographic, lifestyle, and clinical confounders. Importantly, our analysis systematically evaluated the associations between dietary inflammatory potential, CKM severity, and long-term mortality outcomes—offering a comprehensive perspective across disease development, progression, and prognosis.

Nonetheless, several Limitations should be acknowledged. First, dietary intake was assessed via self-report, which is subject to recall bias and misreporting. Secondary, although the DII has been widely validated, its calculation in this study was Limited to 28 of the original 45 food parameters due to NHANES data constraints. Notably absent were several bioactive components with recognized anti-inflammatory properties, such as flavonoids, certain herbs and spices. The omission of these components may have led to a partial representation of the diet’s inflammatory potential, potentially overestimating pro-inflammatory dietary exposures. Lastly, the observational design inherently limits causal interpretation. Future research, including well-designed randomized controlled trials, is warranted to determine whether dietary interventions that lower DII scores can effectively mitigate CKM progression and reduce long-term mortality risks.

## Conclusion

In conclusion, higher DII scores are strongly associated with more advanced CKM syndrome and elevated risks of all-cause and cardiovascular mortality. These associations vary across subpopulations, highlighting the importance of personalized risk assessment. Our results support the inclusion of dietary inflammation measures like the DII in CKM risk prediction tools and public health strategies. Future randomized controlled trials are needed to determine whether reducing dietary inflammatory load can improve outcomes in individuals with or at risk of CKM syndrome. Promoting anti-inflammatory dietary patterns may offer a promising, cost-effective approach to reducing the growing burden of CKM-related morbidity and mortality.

## Data Availability

The data used in this study can be obtained upon request from the corresponding author. Due to privacy and ethical concerns, these data are not accessible to the public.
